# Computational approaches for rational design of proteins with novel functionalities

**DOI:** 10.5936/csbj.201209002

**Published:** 2012-09-28

**Authors:** Manish Kumar Tiwari, Ranjitha Singh, Raushan Kumar Singh, In-Won Kim, Jung-Kul Lee

**Affiliations:** a Department of Chemical Engineering, Konkuk University, 1 Hwayang-Dong, Gwangjin-Gu, Seoul 143-701, Korea; b Institute of SK-KU Biomaterials, Konkuk University, 1 Hwayang-Dong, Gwangjin-Gu, Seoul 143-701, Korea; † These authors contributed equally

**Keywords:** *De novo* protein design, computational protein design, designed therapeutic proteins, metalloproteins, ROSETTA, K* algorithm, DEZYMER, ORBIT

## Abstract

Proteins are the most multifaceted macromolecules in living systems and have various important functions, including structural, catalytic, sensory, and regulatory functions. Rational design of enzymes is a great challenge to our understanding of protein structure and physical chemistry and has numerous potential applications. Protein design algorithms have been applied to design or engineer proteins that fold, fold faster, catalyze, catalyze faster, signal, and adopt preferred conformational states. The field of *de novo* protein design, although only a few decades old, is beginning to produce exciting results. Developments in this field are already having a significant impact on biotechnology and chemical biology. The application of powerful computational methods for functional protein designing has recently succeeded at engineering target activities. Here, we review recently reported *de novo* functional proteins that were developed using various protein design approaches, including rational design, computational optimization, and selection from combinatorial libraries, highlighting recent advances and successes.

## Introduction

Proteins, polymers of amino acids, are the main building blocks and functional molecules of the cell. They are the most multifaceted macromolecules in living systems and have various important functions, including structural, catalytic, sensory, and regulatory functions. The ability of proteins to cluster together to form well-defined structures comprising amino acid sequences make these numerous roles possible. The collection of data regarding protein sequences is rapidly growing, with approximately 6 million entries in Universal Protein Resource (UniProt) knowledgebase at present [1–8]. To completely understand the function of a protein, knowledge of its three-dimensional structure is essential. Unfortunately, experimental structure determination is only possible for a small fraction of these proteins [2, 8–10], with only approximately 2% having experimentally verified structural annotation at present. For the remaining 98%, prediction of the structure is the only alternative. Therefore, the structural characterization of proteins is a major goal in computational biology [1–8].

Advances in molecular modeling have expanded the area of computational protein design, from creating new proteins based on known protein sequences present in nature to designing new proteins that fold into a specific structure or perform a specific function. Before aiming at protein design by using computational methods, one should understand the underlying physical principles governing the folding, stability, and function of a protein. For all these decades, scientists and researchers have been following a perturbation or an alternation-based paradigm in order to determine the functionality of a protein. The method relies on the generation of hundreds and thousands of protein mutants, coupled with selective pressure to identify variants with desired properties. Alternatively, in computational protein design one aims at a design-based paradigm instead of a perturbation-based paradigm. In design-based paradigm, biologists combine design paradigms or methods for problem solving with computational modeling techniques to predict the success of their designs. This paradigm has been effective for the creation and implementation of new ideas and inventions. Design-based paradigm is used for the identification of the boundaries of possible designs and for the elimination of impossible, impractical, inefficient, or otherwise undesirable designs which would have otherwise been difficult to identify using alternation-based paradigm. In a structure-based computational method, a computational or a mathematical framework is constructed by taking into consideration the evolution, function, stability, and functionality of a protein. The designed proteins are then checked experimentally for their specific function. If the designed proteins exhibit all these characteristics, then it can be concluded that the mathematical model or the framework can fundamentally capture the essence of a protein. On the other hand, if the experiments do not work then one can learn from the failures to modify and create a new model, which will ultimately serve the final goal of computationally designing a novel viable protein. Protein design from scratch is thus the most precise way of testing our knowledge on how natural proteins implement their functions.

**Figure 1:**
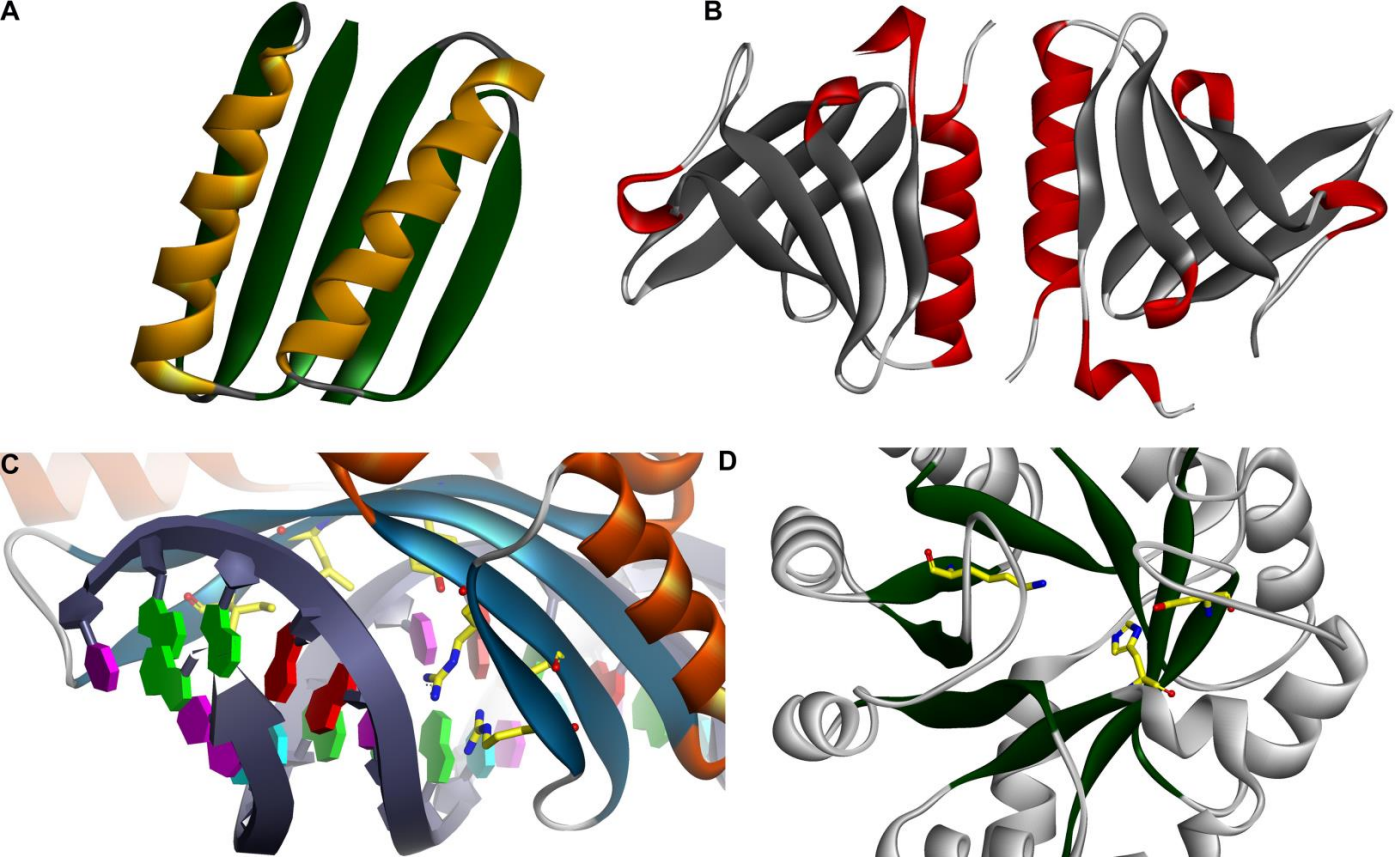
**Computationally designed structures and enzymes.** (*A*) A novel Top7 globular protein fold with atomic-level accuracy [34]. (*B*) Designed SspB adaptor protein [15]. (*C*) Redesigned endonuclease DNA binding [17]. The redesigned enzyme binds and cleaves the redesigned recognition site ∼10,000 times more effectively than does the wild-type enzyme, with a level of target discrimination comparable to the original endonuclease. (*D*) A novel retro-aldol enzyme designed within a TIM-barrel scaffold [20].

Engineering proteins with improved functionality or novel applications has been experimentally achieved by screening of large mutant libraries. However, most of these proteins do not provide quantitative design principles and/or comprehend the structural features that support the desired function. Computational protein design has helped overcome these drawbacks. With reliable structural predictions [11–14], protein stability at the desired conditions, and accurate description of intermolecular interactions (protein–protein interactions [15, 16] and DNA–protein interactions [17]), the technique of computationally designing proteins has been one of the fast-emerging trends in biotechnology and biomedicine. Furthermore, computational protein design has attained significant breakthrough, for example, in the design of novel biocatalysts [18–21] and biosensors for non-natural molecules, redesign of proteins with improved binding affinity [22], redesign of proteins with greater binding specificity [16, 23, 24], and design of proteins capable of binding non-biological cofactors [25] (Figure 1).

Enzyme design presents a huge challenge, not only in the *de novo* design of catalysts for which no natural counterparts are known, but also in the design of multipurpose enzymes, which may have a wide range of biotechnological applications in fields, such as industrial organic synthesis and metabolic engineering [26–29]. This review mainly discusses the strengths and recent successes of computational protein design approaches. We also summarize advancements of design methodology and the application of protein design strategies over the past few years. Other recent reviews can provide additional backgrounds and perspective [30–33].

## Rational computational design

The creation of biocatalysts from scratch enables scientists and engineers to build synthetic enzymes for a series of different chemical reactions, e.g., retro-aldol reaction [20] and Kemp elimination [21]. It also presents a testing ground for our fundamental understanding of the complexities of protein structure and function. Computational protein design starts with the coordinates of a protein main chain and uses a force field to identify sequences and geometries of amino acids that are optimal for stabilizing the backbone geometry [35]. Even for small proteins, the number of possible sequences far exceeds that which can be thoroughly searched. The development of powerful search algorithms to find optimal solutions has provided a major stimulus to the field [36]. Computational protein design requires correlation of structural predictions and experimental stability. Artificial enzymes have been developed with varying degrees of computational involvement, which includes *de novo* enzymes, where both the protein topology and the active site are built from scratch [20, 34, 37, 38].

### De novo active-site design

The introduction of amino acid residues in the form of active site residues into the existing scaffolds is essential for computationally designed enzyme catalysis. These active site residues of the enzymes are responsible for enhancing the chemical reactions by lowering the activation barrier via stabilization of the transition state [39]. Accurate modeling of important forces in the active site requires quantum mechanical (QM) calculations [38]. Potential binding pockets capable of binding tightly to the transition state and retaining the desired geometry of the functional groups are identified within different protein scaffolds. Using geometry-based identification, the transition state is matched with the binding site and the position of the transition state and the catalytic side chains are optimized. Finally, the remaining residues for tight binding of the transition state are designed and the designs are ranked on the basis of transition state binding energy and catalytic geometry. Although the simultaneous design of structure and catalysis promises to broadly expand the scope of artificial enzymes, this area is still in its infancy.

**Figure 2:**
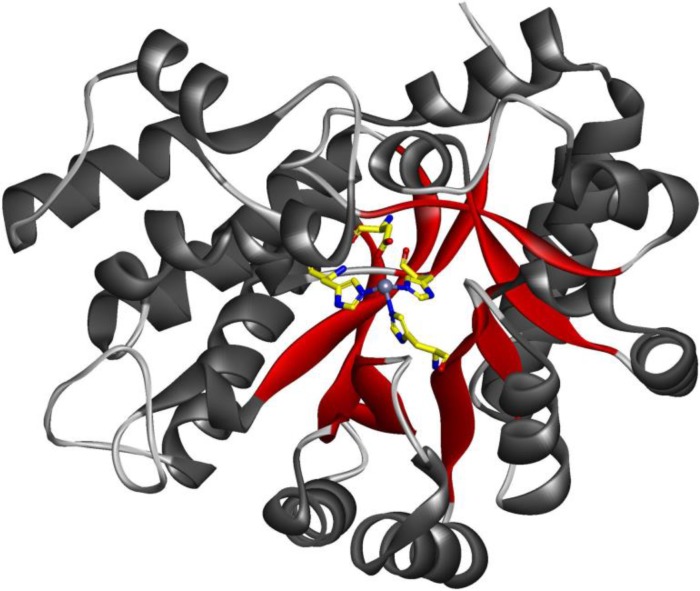
**Computational design of an organophosphate hydrolase.** Engineered zinc-containing mouse adenosine deaminase PT3.1 design crystal structure, with catalytic residues in yellow [49].

Computational techniques have been used to design novel metal binding sites into proteins [40–42]. Nascent metalloenzymes with a variety of oxygen redox chemistries have been generated by leaving one of the primary coordination spheres of the metal unligated by the protein [43, 44]. The diverse and powerful chemistry of metals makes metalloprotein design a promising approach to enzyme design [45]. Early pioneering work included the modeling of iron with one primary coordination sphere ligated with dioxygen, and a number of metalloproteins were designed in the thioredoxin fold [44]. These proteins were experimentally shown to bind iron and catalyzed a variety of oxygen chemistries. A high-energy state of histidine-catalyzed *p*-nitrophenyl acetate (PNPA) hydrolysis as a series of side chain rotamers was modeled by Bolon and Mayo [18]. They had followed a method analogous to that used for the design of catalytic antibodies. *Escherichia coli* thioredoxin [46] was selected as a scaffold because of its favorable expression properties, thermodynamic stability [47], and successful history in computational design [44]. A composite side chain composed of the histidine covalently linked to PNPA was introduced and sampled conformationally around accessible bond rotations in order to computationally model this reaction. To facilitate substrate binding and recognition, amino acids adjacent to the His-PNPA site were allowed to mutate to alanine. The conformations of His-PNPA and surrounding side chains were optimized using Dead End Elimination [48]. This was followed by the synthesis of the top two scoring candidates, protozyme design (PZD) 1 and PZD2. PZD2 demonstrated significant rate enhancements over the uncatalyzed reaction and saturation kinetics with increasing substrate concentration. Although initial extension of these computational methods to the design of a triose phosphate isomerase turned out to be unsuccessful, many important ideas put forth in these studies were incorporated into the recent, successful design of chemically ambitious artificial enzymes. Recently a strategy was devised to design an organophosphate hydrolase starting from a functionally diverse set of mononuclear zinc-containing metalloenzyme scaffolds [49]. For the computational design of organophosphate hydrolysis activity, a dinuclear bacterial organophosphate hydrolase, a zinc-containing enzyme, was selected as the template. Computational design of mononuclear zinc-containing active sites successfully identified a set of mutations in an adenosine deaminase that confer on it the target organophosphate hydrolysis activity (Figure 2). It was found that only four simultaneous mutations were required for the emergence of organophosphate hydrolysis activity in the deaminase. The engineered zinc-containing mouse adenosine deaminase catalyzed the hydrolysis of a model organophosphate with a catalytic efficiency (k_cat_/K_m_) of ∼10^4^ M^-1^ s^-1^, representing a net increase in activity greater than 10^7^-fold.

In parallel with computational advances, active-site designs continue to progress using rational, intuition-based strategies. Using protein and substrate engineering, esterase activity was successfully introduced into human carbonic anhydrase (HCAIII) [50]. The affinity of HCAIII for benzenesulfonamide-containing molecules was used to model a substrate such that the scissile bond was positioned within a cleft in the protein. Grafting a His dyad from previous *de novo* helix-loop-helix designs resulted in an HCAIII variant with enhanced esterase activity over wild-type. The general application of these approaches promises interesting future results, including the ability to design proteins to catalyze reactions that are inaccessible by natural enzymes.

### Computational tools and de novo enzyme design

Computational protein design tools to date have been useful for engineering proteins with a wide range of functions [17, 20, 21, 25, 33, 51, 52]. Many of these successes rely on fixed backbone approaches that maintain the backbone conformations seen in the original high-resolution crystal structures and focus on remodeling only the side chains [53, 54]. Computational protein design programs typically contain two major components: an energy or scoring function to evaluate how well a particular amino acid sequence fits a given scaffold and a search function that samples sequences as well as backbone and side chain conformations. Energy functions for protein-design often contain a combination of physically-based and knowledge based terms [55].

Exceptional progress is seen in *de novo* design of enzymes catalyzing a chemical reaction for which a natural biocatalyst does not exist. Researchers have devised different computational tools to assist designing and engineering of proteins with desired catalytic properties aiming to improve the catalytic efficiency of the designed biocatalysts. Programs like METAL SEARCH [36, 42, 56, 57], DEZYMER [45], ORBIT [58], and ROSETTA [59] have laid a strong foundation and launched the development of *de novo* design of enzymes. METAL SEARCH uses an “on-the-fly binning” algorithm. Binning can be defined as a process of mapping continuous values into categorical values or bins. Binning can amplify data effects and can also reduce the effort required for exception detection by providing a sampling approach. METAL SEARCH helps design tetrahedrally coordinated metal binding sites in proteins of known structure. The program assumes fixed backbones, uses rotamers in the initial stages of the search, and uses simple geometric criteria for evaluating potential sites. The program specifies the 4 residues that might form tetrahedral sites using the backbone coordinates of a protein if wild-type amino acids were replaced by cysteine or histidine. The program has been used for the introduction of zinc binding sites in the designed 4-helix bundle protein a4 and in the B1 domain of streptococcal protein G, and in both cases, the tetrahedral coordination of a bound metal ion has been confirmed. DEZYMER, on the other hand, is a much more versatile computer program than METAL SEARCH, and helps design metal sites in proteins of known structure. DEZYMER is a molecular model building computer program that builds new ligand binding sites into a protein of known 3D structure by altering only the sequence and the side-chain structure of the protein, leaving the protein backbone folds intact by definition. This program enabled computer-aided modeling of sites with pre-defined geometry, providing a general method for the design of ligand-binding sites and enzyme active sites, which can then be tested experimentally. Using a crystal structure as the starting point the program can help maximize the stability of a target state by optimizing the side chain metal-ligand geometries.

Dahiyat and Mayo, via an automation algorithm called ORBIT (optimization of rotamers by iterative techniques), introduced a cyclical protein design strategy by coupling theory, computation, and experimental testing. By using a rotamer description of the side chains, they implemented a fast discrete search algorithm, based on the Dead-End Elimination (DEE) theorem, to rapidly find a globally optimal sequence in its ideal geometry from the vast number of possible solutions. DEE is a powerful algorithm capable of reducing the search space for structure-based protein design by a combinatorial factor. By using a fixed backbone template, a rotamer library, and a potential energy function, DEE identifies and prunes rotamer choices that are probably not part of the Global Minimum Energy Conformation (GMEC), effectively eliminating the majority of the conformations that must be subsequently enumerated to obtain the GMEC [48, 60]. Since the discovery of the DEE algorithm in 1992 several major theoretical and practical improvements have matured the method as a novel and promising tool in the fields of protein modeling and design.

ROSETTA, a milestone for protein design, is a suite of computational tools developed in the laboratory of David Baker. The most widely used computational protein design tool, Rosetta was originally developed for *de novo* fold prediction [11, 12, 61]. But it has been expanded to include methods for design, docking, experimental determination of structure from partial datasets, protein-protein interaction design and prediction, enzyme design, RNA structure prediction and protein-DNA interaction prediction and design [17, 20, 21, 25, 51, 52]. The Rosetta *de novo* enzyme design protocol has been used to design enzyme catalysts for a variety of chemical reactions, and in principle can be applied to any arbitrary chemical reaction of interest. The process has four stages: i) choice of a catalytic mechanism and corresponding minimal model active site, ii) identification of sites in a set of scaffold proteins where this minimal active site can be realized, iii) optimization of the identities of the surrounding residues for stabilizing interactions with the transition state and primary catalytic residues, and iv) evaluation and ranking the resulting designed sequences. Stages two through four of this process can be carried out with the Rosetta package, while stage one needs to be done externally [62]. The code is developed by the RosettaCommons. This working collaborative is composed of over 15 academic groups and thus the code is being applied to a very wide diversity of problems [63]. Recently, ROSETTA was used to develop artificial enzymes that catalyzed retro-aldol reaction [20] and Kemp elimination [21]. These designs were impressive in the extent to which the relationship between structure and reactivity was modeled and characterized.

Kemp elimination and retro-aldol reactions are considered *milestones* in the field of biocatalyst design. Kemp elimination is a model reaction for proton transfer from carbon. Seven rounds of random mutagenesis and shuffling resulted in an enzyme named KE07 (PDB accession code 2RKX) showing a >200-fold increase in catalytic efficiency and >1000 catalytic cycles exhibiting multiple turnovers. KE07 is based on the triose phosphate isomerase barrel scaffold of the *Thermotoga maritime* derived thermostable imidazole-3-glycerolphosphate synthase (HisF). Some of the mutations introduced during directed evolution were localized in the residues adjacent to designed positions; some changed flexibility of the region neighboring the active site; or adjusted pK_a_ of the catalytic residues. The position and functional role of these mutations provide important insight into the strength and shortcomings of current designs, which need to be understood in order to match efficiency of natural catalysts in the future. On the other hand, retro-aldol reactions targeted retro-aldolase as biocatalysts and involved the breaking of carbon–carbon bonds in a non-natural substrate. The design required implementation of hashing methodology in ROSETTA to improve the algorithm in order to meet the requirements for a multistep reaction. The designs spanned a broad range of protein folds and 32 out of 72 experimentally characterized designs showed detectable retro-aldolase activity.

Though Rosetta has been shown to be capable of designing active enzymes in various cases [20, 21, 51], in each case the best designed proteins only had very modest activity, while many of the designs tested had no activity at all. Thus, while this protocol constitutes a powerful tool in the development of novel catalysts, success is by no means guaranteed [62]. In spite of the remarkable success, several shortcomings and potentials for improvement exist, some of which have been briefly summarized in this review. i) To increase the quality of designs, it is beneficial to include as many interactions in the theozyme as possible, and concurrently run matching for as many scaffolds as possible, ii) The enzyme design protocol so far only considers one state of the reactant, or one snapshot of the reaction trajectory. This means that Rosetta will try to design a sequence that optimally stabilizes this state, while ignoring the other states that also occur along the reaction coordinate. Finally, ranking and selection of designs could be improved by the development of faster more thorough computational examination methods [62]. Recently Jacak, R. et al. also pointed out in their study that that in many cases the Rosetta scoring function fails to prevent large hydrophobic clusters on the surface of proteins, even though the overall amino-acid composition of the protein surface is not significantly different from other soluble proteins [64]. Despite the limitations listed above, what distinguishes Rosetta's computational approach is that it is capable of generating catalytic activity from an inert scaffold, whereas most experimental methods, such as directed evolution approaches, rely on an existing catalytic activity as a starting point.

Several other novel algorithms or methods were also developed for *de novo* design of enzymes. A method for designing a new *de novo* protein was developed where one could search suitable scaffolds directly in the Protein Data Bank (PDB) [65]. Triose phosphate isomerase enzyme was used for the authentication of this method. Another novel algorithm based on dead-end elimination was useful for identifying minimized global minimum energy conformations and for the filtering of ensemble-based scoring [66]. A fast and precise algorithm that identified point mutations responsible for changing the net charge of the enzyme was also developed. This was done by bearing in mind that the enzyme maintained its fold and activity for redesigning. This resulted in the change in the pK_a_ values of the catalytic residues placed into the putative catalytic sites [67]. The initial *de novo* designs could be additionally fine-tuned by simulations employing transition states [68, 69].

Another open-source, freely-available computational structure-based protein design suite of programs OSPREY (Open Source Protein REdesign for You) developed in the lab of Bruce Donald at Duke University identify protein mutants that possess desired target properties (e.g., improved stability, switch of substrate specificity, etc.). OSPREY can also be used for predicting small-molecule drug inhibitors. OSPREY incorporates set of several different algorithmic modules for structure-based protein design, including a number of powerful Dead-End Elimination (DEE) algorithms and the ensemble-based K* algorithm for protein-ligand binding prediction. This suite allows the incorporation of continuous protein side-chain and continuous or discrete backbone flexibility, while maintaining provable guarantees with respect to the input model (input structure, rotamer library, and energy function) for a given protein design problem. OSPREY also includes many extensions and improvements to the DEE framework (e.g., minDEE, iMinDEE, K*, DACS, BD, BRDEE). These extensions improve efficiency and allow the modeling of molecular flexibility. OSPREY includes the K* module, which is a provably-good e-approximation algorithm for computing binding constants (*K*_D_) over molecular ensembles of the bound and unbound states of a protein:ligand complex using minimized DEE/A* (namely, minDEE/A*/K*) [70, 71]. In 2009, Chen et al. reported a computational structure-based redesign of the 65-kDa phenylalanine adenylation domain of the nonribosomal peptide synthetase (NRPS) enzyme gramicidin S synthetase A (GrsA-PheA) for a set of noncognate substrates [70]. They applied the K* algorithm [66, 72] to predict mutations to the active site of GrsA-PheA to switch the enzyme specificity from the wild-type Phe toward the target noncognate substrates Leu, Arg, Glu, Lys, and Asp [70] and to predict mutations in dihydrofolate reductase from methicillin-resistant *Staphylococcus aureus* (MRSA) [73].

Amy E. Keating and her coworkers at Massachusetts Institute of Technology have been working on how the interaction properties of proteins (alpha-helical coiled coils and Bcl-2 family proteins) are encoded in their sequences and structures. Multicoil2, an algorithm predicting both the location and oligomerization state (two versus three helices) of coiled coils in protein sequences was developed by the Keating's group. It combines the pairwise correlations of the previous Multicoil method with the flexibility of Hidden Markov Models (HMMs) in a Markov Random Field (MRF). This new method significantly improves oligomer-state prediction, as well as coiled-coil detection, over the algorithms Multicoil and Paircoil2 [74, 75]. The performance of Multicoil2 is especially notable in the twilight zone of sequence identity, where HMM profile-based methods typically fail [76]. Recently Joe DeBartolo et al. at the Keating's laboratory used a combination of experimental assays and computational models to explore Bcl-2 homology 3 (BH3) peptide interactions with Bcl-2 family receptors [77]. They evaluated a novel structure-based protein–protein interaction statistical potential called STATIUM that can score interactions of BH3-like peptides with all five Bcl-2 receptors and is rapid enough to evaluate data sets containing more than 106 sequences in less than 1 s. The very general structure-based STATIUM model shows remarkably good performance compared to the experimentally derived position-specific scoring matrix (PSSM) models. STATIUM demonstrates great potential for evaluating candidate protein–protein interactions and can be used to complement other structure-based modeling techniques such as Rosetta, DFIRE, or MM/PBSA that require accurate construction of all atom models [78–81].

A recent milestone in the field of computational enzyme design has been the *de novo* design of a Diels-Alderase (DA) [51]. David Baker and his group had previously used the Rosetta computational design methodology to design novel enzymes [20, 21] that catalyze bond-breaking reactions. Using DA reactions, they have tried to establish bimolecular bond-forming reactions. The concept required carbon-carbon bond formation between two separate substrates, catalyzing an intermolecular Diels-Alder reaction that requires the concomitant binding of two substrates in their proper relative orientation in order to accelerate the reaction and impart stereoselectivity. Quantum mechanical (QM) simulation to create a comprehensive theozyme library (∼10^19^ variants), which was fitted into a library of protein scaffolds by the RosettaMatch software, was employed. The ∼10^19^ active site configurations were reduced to about 10^6^ possible protein scaffolds. Optimization of these protein scaffolds led to 84 protein designs. The 84 possible proteins were then synthesized within *E. coli* and then tested for catalytic behavior in the Diels-Alder reaction, resulting in the identification of two candidates having detectable DA activity. The catalytic efficiency of these two synthetic DAs matched the performance of catalytic antibodies raised for Diels-Alder cycloadditions and exhibited stereoselectivity and substrate specificity. X-ray crystallography confirmed that the structure matched the design for the most active of the enzymes, and binding site substitutions reprogrammed the substrate specificity. Recently, an increased DA activity through backbone remodeling was achieved using the computer game called Foldit [82–85]. The active-site loops of a computationally designed enzyme, DA_20_10 [51], that catalyzes the Diels-Alder reaction, were remodeled. DA_20_10 catalyzed the well-studied reaction between 4-carboxybenzyl trans-1,3-butadiene-1-carbamate (diene) and N,N-dimethylacrylamide. A 24-residue helix-turn-helix motif, including a 13-residue insertion, was generated after several iterations of design and characterization, that increased the enzyme activity to >18-fold. Using this game Baker and coworkers aimed to exploit human problem-solving skills to improve the limitations of computer-designed proteins, which otherwise computers cannot solve alone.

The *de novo* designed enzymes are functional but do not match natural catalysts in their efficiency. Their catalytic efficiency is still many orders of magnitude below the natural enzymes [86]. Current models still tend to lag behind laboratory-evolved variants in catalytic performance. While some experimental optimization is possible by directed evolution, refinements in the design algorithm will likely yield further improvements in the accuracy of structure predictions and hence provide superior catalytic performance. Separately, the integration of protein dynamics in future simulations might deliver additional functional enhancement and at the same time provide an excellent testing ground for assessing its relevance to biocatalysis.

## Engineering novel metalloproteins

Metalloproteins are proteins containing metal atoms or clusters. They are responsible for a diverse range of important biological functions and are involved in all vital cellular activities. The metal, contained within a metalloprotein, may be an isolated ion or may be coordinated with a non-protein organic compound. For example, hemoprotein containsporphyrin. Alternatively, the metal is co-coordinated with a side chain of the protein and an inorganic non-metallic ion, such as in the case of iron-sulfur clusters. Metalloenzymes occur in all six Enzyme Commission (EC) classes, accounting for 44% of oxidoreductases, 40% of transferases, 39% of hydrolases, 36% of lyases, 36% of isomerases, and 59% of ligases [87]. Metal ions add new functionality to proteins and help catalyze some of the most difficult biological reactions. Furthermore, with their varying redox states and geometric arrangements, metal ions enhance protein reactions. Probably for these reasons, metal-binding proteins account for about 50% of all proteins [88].

A promising approach to metalloprotein design is the knowledge and utilization of the diverse and powerful chemistry of metals [89]. Early pioneering work included the development of a computational method to identify protein sites capable of using side chains to complex metal atoms [45]. A number of metalloproteins were designed in the thioredoxin fold by modeling iron with one primary coordination sphere ligated with dioxygen [44]. These proteins were experimentally shown to bind iron and catalyzed a variety of oxygen chemistries. One such class of these metal binding proteins is the ‘Duo-Ferri’ (DF) series of maquettes which was developed to mimic di-iron proteins [90]. The DF maquettes bind two iron atoms and can also bind other metals ions (Zn, Co and Mn) with the stoichiometry of two ions per protein [90, 91].

The emphasis of designing as a tool has been expanded from the understanding of important characteristics or functionality of naturally occurring metalloproteins, to the design of functionally active novel artificial metalloproteins. However, designing of metalloproteins has proven to be more challenging than the design of non-metalloproteins. Most metal-binding sites are highly chromatic and display distinctive magnetic properties, making it easier to characterize the designed metalloprotein using metal-based spectroscopic techniques thus shortening design cycles. Therefore, the field of metalloprotein design has enjoyed much success recently, owing to advances in biophysical, computational and structural biology [92]. Therefore, designing and engineering novel metalloproteins is an important test of our ability to design proteins. One of the most important developments in the designing of metalloproteins has been the design and engineering of a novel metal-binding site into a native protein with a characteristic scaffold. This achievement has given rise to novel proteins catalyzing some of the most complicated biological reactions. Another advantage of choosing metal-binding sites as targets for protein design is the rich spectroscopic data available for evaluating the design process.

Extensive study of metalloproteins has been carried out using biochemical techniques such as site-directed mutagenesis [93–97]. The loss of function accompanied by certain mutations allows the identification of residues essential for function. Although serving a different purpose, the same mutagenesis techniques can be used in metalloprotein design to impart new function into a protein scaffold by introducing residues that bind metal ions. One of the most effective approaches in the design and engineering of novel metalloproteins is the redesign of existing metal-binding sites to new sites possessing totally different structural and functional properties. This approach can be best illustrated using heme proteins. Heme proteins catalyze a variety of reactions ranging from electron transfer, small molecule transport and sensing, to oxygen activation. Redesign of heme proteins, from one type into another, provides a test of the known factors governing the structure and function of a heme protein, and allows the direct comparison of two different heme proteins in the same framework. Heme proteins have been redesigned by varying the axial or proximal ligand, by redesigning the distal side of the heme and/or by redesigning one type of heme protein into another type.

On the basis of sequence, mechanistic, and structural information, and a novel SIAFE (simultaneous incorporation and adjustment of functional elements) process, the active site of glyoxalase II αβ/βαa metallohydrolase scaffold (GlyII) was reconstructed to bind and catalyze the hydrolysis of a typical substrate for metallo β-lactamase (MBL), cefotaxime [98]. In order to achieve β-lactamase activity the metal-binding sites of the GlyII had to undergo complex redesigning. In addition to heme, other metal ions/cofactors have been engineered into helical bundles by introducing metal-binding ligands at specific locations to mimic those in native proteins. Examples are the Cys_2_His_2_ ligand set found in zinc-finger proteins [99] and the His_3_ set in carbonic anhydrase [100].

Introduction of metal-binding sites into a protein location where no native metal-binding site is found has provided insight into the structural features common to the metal-binding sites of template and target proteins. Using structural homology between the template protein (which contains no metal ions) and the target metalloprotein, metal-binding sites can be introduced into the template protein at the positions corresponding to those in which they are found in the metalloprotein. Using this strategy, new Zn(II)-binding sites were introduced into charybdotoxin [101] and retinol-binding protein [102] to mimic carbonic anhydrase. These developments in the field of metalloproteins lead to the design of dinuclear metal-binding sites or metal clusters. For example, the designing of Cu_A_ centres into the cupredoxinsazurin [103, 104] and amicyanin [105, 106], in which the copper-binding loop of a cupredoxin was replaced by the corresponding loop in cytochrome *c* oxidase (COX; also known as C*c*O), which has similar structural homology. A series of His_3_Fe sites were introduced to thioredoxin in various environments, classified as grooves, shallow pockets and a deep pocket, allowing the effect of the protein microenvironment on “nascent” enzymatic activity to be studied [43, 44].

A sequence-homology modeling and molecular dynamics simulation was employed to assemble the presumptive active site metal complex of nitric oxide reductase (NOR) in whalesperm myoglobin (Mb) [107]. NOR is a metalloenzyme in the denitrification pathway of anaerobic bacteria, and is a key enzyme in the nitrogen cycle that is critical for all life [108]. Difficulties in obtaining the enzyme in high yield, and the lack of a three-dimensional structure, have hampered structural and mechanistic studies of NORs. A strong model system to explore the spectroscopic properties, and to validate the catalytic function of the hypothetical metal complex in the Mb scaffold, was established using the assembly of the proposed catalytic site, consisting of a heme and a putative Fe_B_ site. A hydrophobic pocket near the heme cofactor was subjected to remodeling in order to establish the new non-heme Fe^2+^ binding site. The remodeling was done by introducing three histidines and one glutamate, predicted to be ligands in the active site of NOR, into the distal pocket of Mb. A crystal structure of the designed protein confirmed that the minimized computer model contained a heme/non-heme Fe_B_. The designed protein also exhibited NO reduction activity, and thus modeled both the structure and function of NOR, offering insight that the active site glutamate is required for both iron binding and activity. The designed protein served as an excellent model for mechanistic studies of NOR. Engineering of the metal-binding sites of metalloproteins, and study of native enzymes, has enabled identification of the structural features that are necessary to confer the structure and function of these enzymes.

**Figure 3:**
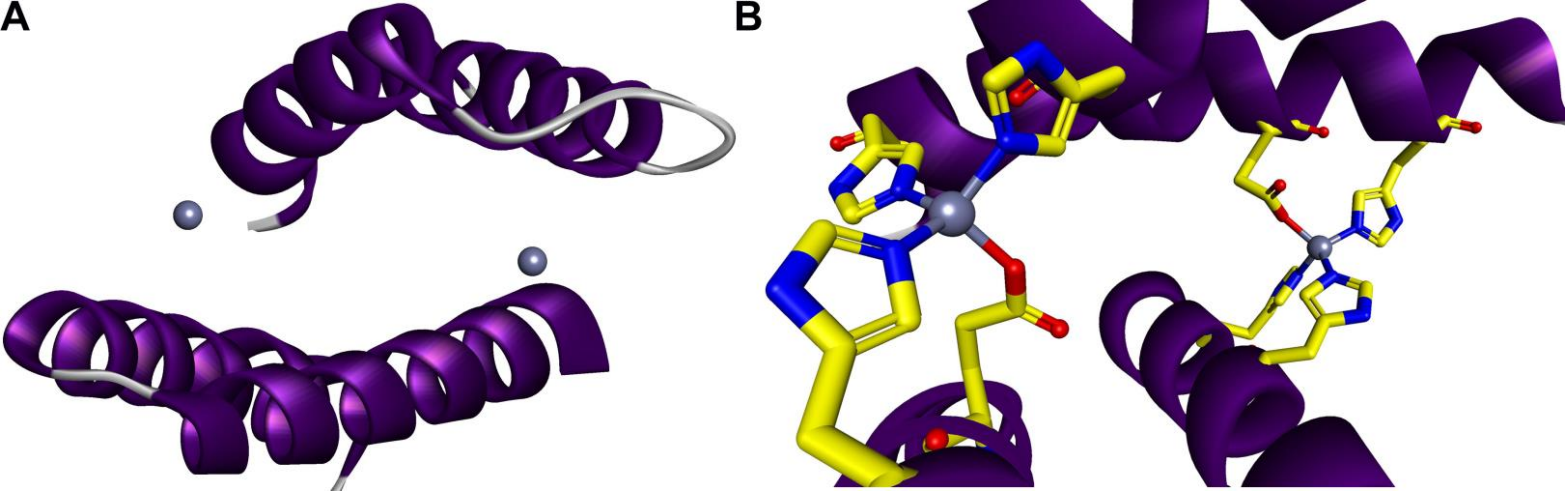
**Computationally designed protein-protein interactions with high affinity and desired orientation.** (*A*) The symmetric homodimer design with two interface zinc sites each coordinated by four histidines at i, i + 4 positions on each helix [112]. A Rosetta-based approach for the rational design of a protein monomer to form a zinc-mediated, symmetric homodimer. Incorporating metal-binding sites at the target interface may be one approach for increasing affinity and specifying the binding mode. (*B*) Metal interface design, named MID1 (NESG target ID OR37), forms a tight dimer in the presence of zinc (MID1-zinc) with a dissociation constant <30 nM.

Using a “metal-first” approach [109, 110], a four-helix bundle protein was designed to bind four-iron four-sulfur (Fe_4_S_4)_ in its hydrophobic core. This is particularly noteworthy given that natural Fe_4_S_4_-binding proteins are not a-helical and generally bind the ligand in flexible loops [111]. Recently, Kuhlman and his group devised a strategy of introducing a metal binding site at the target interface in order to promote protein interaction (Figure 3). In order to pursue their goal, they computationally designed a metal-mediated homodimer MID1 (metal interface design 1) with high affinity and orientation preference. The steps involved for the design of the symmetric metal-mediated interface are: i) examination of two-residue cysteine/histidine zinc binding sites using the RosettaMatch algorithm. During this step about 600 monomer scaffold surfaces were scanned, ii) grafting of all pairs of two-residue zinc binding sites for a given scaffold onto the surface, and the conversion of a monomer to a C_2_-symmetric dimer by rotation. This step resulted in the identification of 500,000 designable starting structures among 600 scaffolds using their Rosetta protocol, iii) using Monte Carlo simulated annealing, iteration of symmetric interface design with symmetric backbone minimization, and iv) finally, identification of best design models on the basis of two primary metrics: computed binding energy, excluding contribution from zinc (ΔG_bind_), and binding energy per unit of interface surface area (ΔG_bind_/ΔSASA). Eight best models obtained were finally subjected to testing. The final computationally designed metal-mediated homodimer MID1 (metal interface design 1), with high affinity and orientation preference, was only successful after considering the crystal structures of previous iterations in the design process. In the absence of metal, the MID1 design dimerized only weakly and with two types of nonspecific orientations. In the presence of metal, the desired binding orientation was achieved with high affinity, despite minor discrepancies at the atomic level between the computational model and the crystal structure [112]. *De novo* protein design thus provides an attractive approach for modeling the active sites of metalloproteins. The design strategy presented here enabled the conversion of an enzyme in the metallohydrolase superfamily into a new family member with a different catalytic function, providing experimental support for the divergent evolution of mechanistically diverse family enzymes.

## Design and engineering of therapeutic proteins

Computational design holds great potential for the development of new protein-based therapeutics with novel modes of action. The method of systematic and quantitative engineering strategies for protein optimization is now being replaced by computational protein design methods. Antibodies are the predominant class of computationally designed proteins that are used as therapeutics. Rationally designed antibody molecules catalyze numerous chemical transformations, including many that cannot be achieved by standard chemical methods. Using computational methods different hapten [113] and antibody [114] design strategies have been developed. These strategies include a transition state analog approach. However, the catalytic efficiency of the resulting molecules has been low relative to natural enzymes [115].

In a significant advance, catalytic antibodies that utilize a nucleophilic mechanism were selected by reactive immunization and resulted in efficient catalysts [115]. Rather than a transition state analog, a mechanism-based inhibitor was used to elicit the immune response. Antibodies that formed stable covalent attachments to the suicide inhibitor were effectively selected. This method was employed in the selection of an efficient abzyme (catalytic antibody) with a nucleophilic lysine for aldol condensations [116]. The efficiency of this aldolase demonstrates the effectiveness of covalent catalysis. The ability to select for powerful catalytic groups and active sites with high transition-state specificity could theoretically yield more efficient catalysts.

Antibodies have several drawbacks despite their significant clinical success. They are large, which limits their entry into tumors and tissues, require expensive manufacturing and handling facilities, and often cause undesired effector functions. Although smaller antibody fragments have been developed, they are often associated with weaker binding than the intact antibody, they can exhibit lower stability, and they might expose immunogenic epitopes that were previously masked [117]. This has led to the development of ligand- and receptor-based agonists or antagonists with therapeutic potential.

**Figure 4:**
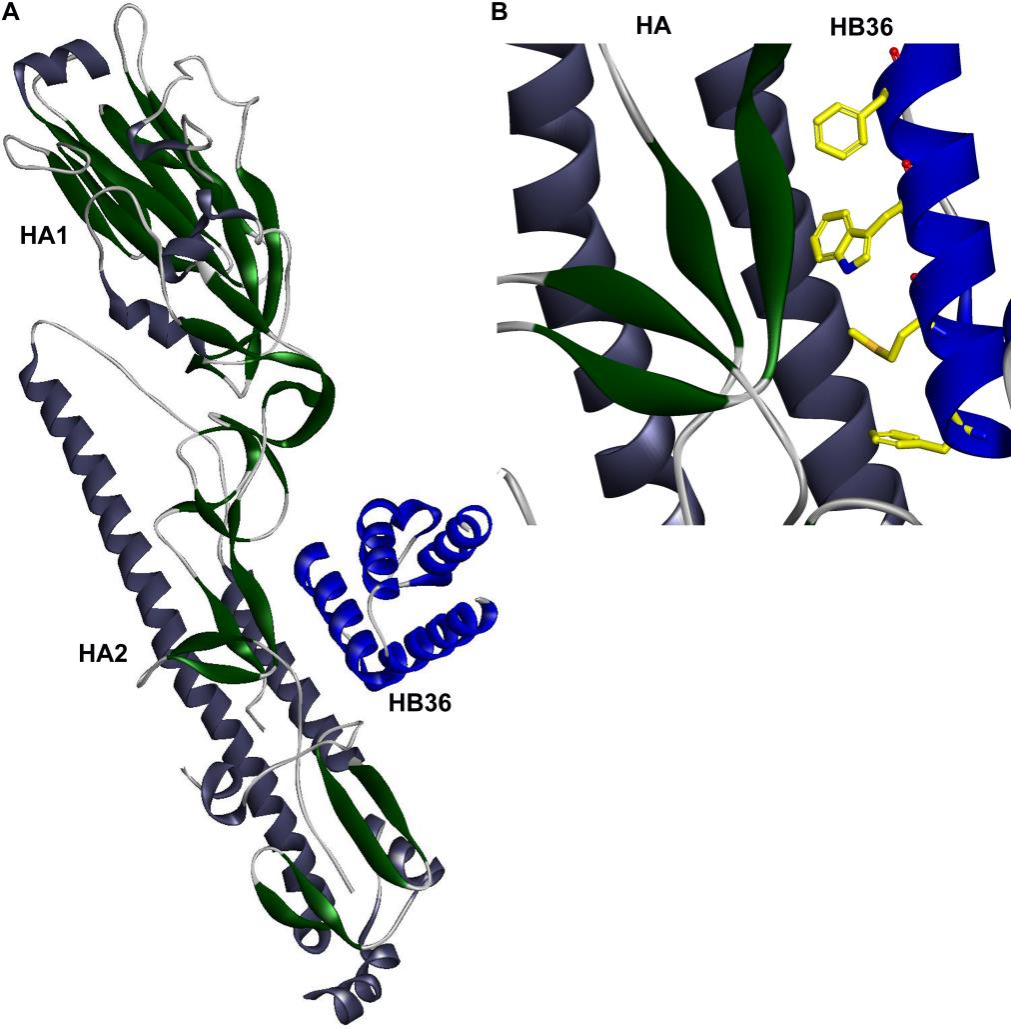
**Design of novel binding proteins.** (*A*) Crystal structure of HB36.3-SC1918/H1 complex . (*B*) Close up view of SC1918 HA-HB36.3 interface [52].

A program that utilizes the information embedded in a protein structure to optimize the function of a protein, including its activity, binding affinity and specificity, stability, expression level, and potency, was developed by Xencor. Xencor's Protein Design Automation (PDA™) technology [58, 118–121] couples computational design algorithms, which generate quality sequence diversity, with experimental high-throughput screening to discover proteins with improved properties. In order to capture the relationships between protein sequence, structure and function accurately the computational program uses atomic level scoring functions, side chain rotamer sampling and advanced optimization methods. Another method was used to design proteins that bind a conserved surface patch on the stem of the influenza hemagglutinin (HA) from the 1918 H1N1 pandemic virus [52]. Two of the designed proteins (HB36 and HB80), after affinity maturation, were found to bind H1 and H5 HAs with low nanomolar affinity. HB80 was demonstrated to inhibit the HA fusogenic conformational changes induced at low pH. Such designed binding proteins may be useful for both diagnostics and therapeutics (Figure 4).

The most effective way to design protein drugs are using computational methods in conjunction with functional screening techniques. *In silico* methods can explore much larger portions of sequence space than can be accessed experimentally, and can be used to design targeted libraries that are enriched in functional sequences [122–124]. Although computational design holds great potential for the development of new protein-based therapeutics with novel modes of action, many challenges remain.

## Conclusions

Computationally designed proteins offer promise in many areas of research, from basic biology to application in the fields of industrial organic synthesis and biomedicine. There has been remarkable progress in the field of rational enzyme design. This field has evolved from the design of proteins with improved features, such as thermostability, catalytic activity, better metal affinity, substrate specificity, or stereoselectivity, to the design of novel proteins and folds *ab initio*. The field has made exciting progress, designing proteins with new structures and functions. In early 1998, a novel sequence that folded into a naturally occurring zinc finger structure was computed. Later in 2003, an exceptionally stable protein called Top7, which has a sequence and structure unrelated to any known protein, was designed using ROSETTA. This achievement, of having the power to create a brand new protein, encouraged the scientific community to design novel proteins with atomic-level accuracy. Recently in 2010, a non-natural aldolase capable of catalyzing reactions of non-natural substrates was designed. The aldol reaction constitutes one of the most powerful tools for the formation of carbon-carbon bonds both in nature and in the laboratory. In 2012, the most recent accomplishment in the field of computational design approach was the achievement of an increased Diels-Alderase (DA) activity through backbone remodeling, which was achieved using a game called Foldit. Table 1 lists a number of different enzymes that have been evolved using the computational protein design approaches over the past few years.

**Table 1:** Summary of computationally designed biocatalysts with novel functions

Target	Protein design goal	Methodology		Metric(s)	Conclusions	Ref.

		Computational	Experimental			
Diels-Alderase	Biocatalyst for intermolecular Diels-Alder reaction (Novel catalysis)	QM/MM simulations, RosettaMatch and Design software	Site-directed mutagenesis	*k*_cat_ = 0.036 min^-1^; *k*_cat_/*K*_m_ = 0.455 M^-1^ s^-1^	Stereoselective Diels-Alderase, functional performance matches catalytic antibodies	[51]
Kemp eliminase	Novel catalysis	*De novo* design via Rosetta	Directed evolution	*k*_cat_ = 1.37 s^-1^, *k*_cat_/*K*_m_ = 2590 M^-1^ s^-1^	>200-fold increase in *k*_cat_/*K*_m_	[21]
Nitric oxide reductase	Reconstitute active site of NOR in myoglobin	VMD software (molecular modeling)	Site-directed Mutagenesis	Yield of N_2_O production by Fe(II)-FeBMb was estimated to be ∼30%	Functional model of NOR	[107]
Gramicidine S synthetase A (GrsA-PheA)	Substrate specificity from Phe to Leu, Arg, Lys, Glu, or Asp	K* algorithm (mutagenesis with rotamer library, flexible backbone and dynamic ligand substrate)	Site-directed mutagenesis	*k*_cat_ = 0.85 min^-1^, *k*_cat_/*K*_m_ = 159.86 mM^-1^ min^-1^	600-fold specificity shift for Phe→Leu due to changes in *K*_m_-values	[70]
Mouse adenosine deaminase	Hydrolysis of a model organophosphate	Rosetta Design, RosettaMatch	Directed evolution	*k*_*cat*_/*K*_m_ ∼10^4^ M^-1^ s^-1^	Enhanced activity by ∼2,500-fold	[49]
Rab4-binding domain of rabenosyn	Protein monomer to form a zinc-mediated, symmetric homodimer	RosettaMatch	Site-directed mutagenesis	Tight binding (*K*_d_< 30 nM)	Zinc binding leads to a >200-fold increase in binding affinity	[112]

Despite all these major progresses and breakthroughs in the field of enzymology, the designed or engineered enzyme catalysts remain inferior to naturally evolved enzyme catalysts in terms of activity and efficiency. One such example is that of an artificially designed Kemp eliminase. However, the artificially designed enzyme exhibited an improvement in the catalytic efficiency of >200-fold when subjected to directed evolution, which resulted in a protein with multiple mutations. Accurate structure modeling, protein stability, and intermolecular interaction optimization remain the major challenges in the fields of computational protein design. Each of these major barriers has received significant attention in the past few years and many artificial protein designs have been produced as a result. In addition, incorporation of the protein backbone and ligand exchange between the active site and the solvent in the computational design methods will help improve artificially designed proteins. Although there is still a long way to go, with improvements to algorithms and increases in computing power, exciting progress is being made in both prediction and design. The success obtained by rational computational design is extremely encouraging, demonstrating that protein design represents a fundamental tool for understanding protein folding and interaction. Rational computational design promises a great positive impact on both the biotechnology and the therapeutic industry, thus revolutionizing the fields of molecular biology and biomedicine.
